# Doxorubicin-Conjugated Iron Oxide Nanoparticles Synthesized by Laser Pyrolysis: In Vitro Study on Human Breast Cancer Cells

**DOI:** 10.3390/polym12122799

**Published:** 2020-11-26

**Authors:** Iulia Ioana Lungu, Simona Nistorescu, Mădălina Andreea Badea, Andreea-Mihaela Petre, Ana-Maria Udrea, Ana-Maria Banici, Claudiu Fleacă, Ecaterina Andronescu, Anca Dinischiotu, Florian Dumitrache, Angela Staicu, Mihaela Balaș

**Affiliations:** 1 National Institute of Laser, Plasma and Radiation Physics, 409 Atomistilor Street, 077125 Magurele, Ilfov, Romania; iulia.lungu@inflpr.ro (I.I.L.); simona.stroescu@inflpr.ro (S.N.); ana.udrea@inflpr.ro (A.-M.U.); ana.niculescu@inflpr.ro (A.-M.B.); claudiu.fleaca@inflpr.ro (C.F.); 2 Faculty of Applied Chemistry and Materials Science, University Politehnica of Bucharest, 1-7 Gh. Polizu Street, 011061 Bucharest, Romania; ecaterina.andronescu@upb.ro; 3 Department of Biochemistry and Molecular Biology, Faculty of Biology, University of Bucharest, 91–95 Splaiul Independentei, 050095 Bucharest, Romania; madalina.andreea.badea@drd.unibuc.ro (M.A.B.); petre.andreea-mihaela@s.bio.unibuc.ro (A.-M.P.); anca.dinischiotu@bio.unibuc.ro (A.D.)

**Keywords:** drug delivery, iron oxide nanoparticles, doxorubicin, breast human cells, in vitro cytotoxicity

## Abstract

Even today, breast cancer remains a global public problem, with a high mortality rate among women. Nanoparticle (NP) based systems are developed to enhance drug delivery, reducing the toxic effect of medicine molecules. By using iron oxide nanoparticles for cancer treatment, several advantages were highlighted: the ability to target specific locations derived from their magnetic properties and reduced side effects. The aim of this study was to examine on breast cancer cell line the anticancer potential of γ-Fe_2_O_3_ NPs loaded with doxorubicin (DOX) and stabilized with carboxymethylcellulose sodium (CMCNa). The γ-Fe_2_O_3_ NPs were synthesized by laser pyrolysis technique and their nanometric size and crystallinity were confirmed by X-ray diffraction and transmission electron microscopy. The loading efficiency was estimated by using absorption and fluorescence spectroscopy. The DOX conjugated//CMCNa coated γ-Fe_2_O_3_ NPs proved through the biological studies to have a good anticancer effect through the inhibition of tumoral cell proliferation, disruption of the cellular membrane, induction of cell death and reduced effects on normal breast cells. Our data showed that DOX cytotoxicity increases significantly when conjugated with ɣ-Fe_2_O_3_ and ɣ-Fe_2_O_3__CMCNa, a 50% reduction of cancer cell viability was obtained with a concentration around 0.1 µg/mL.

## 1. Introduction

Breast cancer (BC) is the most common type of cancer in the world among women and it remains a challenge even today. For the next years, statistics show that the diagnosis will come out positive for 1 in 8 women, and it was demonstrated that with age, the incidence of BC increases, but after menopause, the frequency is lower [1,2]. In 2018, the World Health Organization estimated that 627,000 women died of breast cancer [3].

Conventional strategies, such as chemotherapy, radiotherapy and surgical resection, either performed individually or in combination, manifest limited therapeutic effects for cancer because these types of treatment are non-specific [4]. The most common class of drugs utilized in BC chemotherapy is anthracyclines, such as doxorubicin (DOX) [5] or epirubicin [6] administrated in early and advanced stages of breast cancer. Recently, there were identified two possible mechanisms of DOX anticancer effect. The first mechanism refers to the DOX property to intercalate into DNA strands, inhibition of topoisomerase II synthesis and resulting relaxed DNA supercoils. After the topoisomerase II complex breaks the DNA double helix, DOX prevents the reassociation of DNA strands and replication process [7]. The second mechanism of DOX represents the ability to generate oxidative stress into cells generating damages in DNA, proteins, lipids and cell membrane [8,9]. In addition to the beneficial effects of treatment, this drug induces cardiotoxicity [5,10].

Many studies focused on finding new anti-cancer drugs with lower cytotoxicity in healthy tissues. In addition, cancer cells can gain resistance to drug treatment, and these resistant cells suppress intracellular accumulation of drugs. In the last years, nanotechnology had a significant contribution to anticancer therapy by nanocarriers, which enhanced drug delivery, improved efficacy and reduced side effects [4,11,12]. 

Initially, researchers focused prevalently on the chemical composition and physical properties of nanomaterials. Many studies have shown that optical and magnetic properties of NPs are the most important aspects for their detection in tumor imaging and diagnosis [13].

Blood vessels present on the tumor surface can allow the penetration of particles with a diameter between 30 and 200 nm. Accumulations inside the cancer cells could be manipulated by variation of the nanomaterials size [13,14,15]. These types of nanocarriers can control the drug target and can be manufactured with different materials or chemical compounds, such as metals, oxides, polymers, carbon, lipids, etc. The most important advantage of NPs is that they can be conjugated with therapeutic agents, or fluorophores in a single formulation. In addition, due to the ability to optimize the size and surface chemistry, NPs can penetrate cancer cells and target a specific receptor or ligand of a cell [12,16]. Besides, the coupling improves drug efficacy and protects it by the internal environment; NPs could be considered a local deposit for drugs [12]. Among the NPs, magnetic ones have drawn much attention due to theirmagnetic susceptibility, biocompatibility and easy control by an external magnetic field, which can release the anticancer agent at a specific site [17] and also can act as Magnetic Resonance Imaging MRI contrast agents [18]. One of the most widely used types of magnetic NPs are iron oxide NPs (IONPs) with superparamagnetic characteristics, colloidal and chemical stability, and high biocompatibility; these properties make them the best candidates, as drug carriers in cancer therapy or in hyperthermia therapy [17,19].

There are many studies on biomedical and bioengineering applications of IONPs [20]. In nature, the three most ubiquitous iron oxides are magnetite (Fe_3_O_4_), maghemite (γ-Fe_2_O_3_) and hematite (α-Fe_2_O_3_) [21,22]. IONPs are inexpensive, biodegradable and non-toxic, being good candidates for drug loading [23]. IONPs can be coupled with therapeutic molecules either by covalent binding or co-capsulating in polymeric matrices. 

To date, several drugs, including DOX and paclitaxel (PTX), loaded on IONPs have been tested for cancer therapy [24,25]. Polymers’ use was proved to be very efficient for IONPs stability in drug formulations. Agents with combined effect for in vivo cancer imaging and therapy, as anti-biofouling polymer-coated IONPs loaded with DOX were reported [26]. Similarly, Jain et al. developed oleic acid-coated IONPs and stabilized them with pluronic [27]. Conjugation efficiency and the mechanisms that determine the release of the drug are important parameters for development of IONPs nanocarriers [25].

Among IONPs, γ-Fe_2_O_3_ demonstrated the highest biocompatibility, thus making them a good choice for utilization as drug carrier in cancer research studies. The efficiency of γ-Fe_2_O_3_ NPs conjugated with DOX was tested in different solid cancers. For example, Plichta et al. showed [28] an increase of the number of dying human cervix carcinoma cells (HeLa line) and human osteosarcoma cells (MG-63 line) under the action of DOX-conjugated polymer-coated γ-Fe_2_O_3_ NPs by 10–20% compared to free Dox. More recently, a significantly decrease of viability of human glioblastoma cells (GaMG line) was reported in the presence of magnetic γ-Fe_2_O_3_ NPs conjugated with DOX in low concentration (10 nM) [29]. In contrast, in A549 lung cancer cells, the PEG-functionalized γ-Fe_2_O_3_ NPs conjugated with DOX induced very high viability, possible due to the limited release of DOX. However, under alternating magnetic field (AMF), NPs exhibited excellent thermal effects thus favoring the release of DOX from γ-Fe_2_O_3_ NPs and destruction of lung cancer cells [30].

The main purpose of this study is to demonstrate the efficiency of ɣ-Fe_2_O_3_ loaded with DOX in breast cancer therapy. The IONPs were synthesized by laser pyrolysis technique using iron pentacarbonyl (Fe(CO)_5_) vapors and synthetic air as iron and oxygen sources, respectively. NPs synthesized by laser pyrolysis come with major advantages. They present narrow distribution for particle size in comparison with other vapor condensation methods, extra small dimensions (a few nm), low degree of contamination with other elements and improved magnetic and structural properties due to the high temperatures used during synthesis in comparison with wet/chemical methods [31].

The nanopowders were analyzed by X-ray diffraction (XRD) and transmission electron microscopy (TEM).

The nanoparticles were stabilized with carboxymethylcellulose sodium salt (CMCNa) and loaded with DOX. The loading efficiency was estimated by using absorption and fluorescence spectroscopy. The conjugation of the drug with NPs was evidenced by FTIR spectroscopy. The DOX conjugated γ-Fe_2_O_3_ NPs suspensions proved a good anticancer effect by the inhibition of tumoral cell proliferation, disruption of the cellular membrane, induction of cell death, and reduced effects on normal breast cells.

## 2. Materials and Methods 

### 2.1. Synthesis of Iron Oxide Nanoparticles

**Figure 1:**
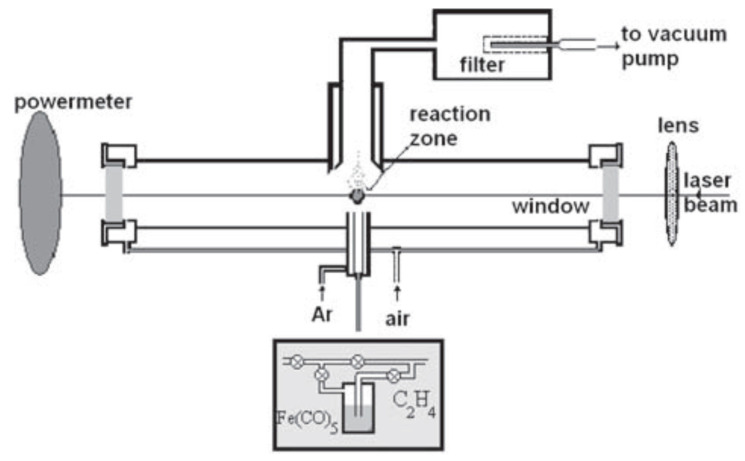
Experimental set-up for obtaining iron oxide nanoparticles [32].

The iron oxide nanoparticles were synthesized by laser pyrolysis technique (Figure 1) using iron pentacarbonyl (Fe(CO)_5_) (Merck, Darmstadt, Germany) and synthetic air as iron precursor and oxidizing agent. Ethylene (C_3_H_4_) (Linde plc., Munich, Germany) was used as an energy transfer agent, also known as sensitizer. The principle of the method depends on the cross-flow configuration that ensures the resonance between the emission line of a CO_2_ infrared laser and the absorption line of the gas sensitizer, subsequently heating the reacting gases through collision energy transfer [32].

Briefly, the focused continuous wave of CO_2_ laser radiation (70 W maximum output power, λ = 10.6 µm) is orthogonally intersected by the reactant flow. The combination between synthetic air (100 sccm) and Fe(CO)_5_ vapors carried by an ethylene flow(100 sccm) entered into the reaction chamber through the central inner tube. The confinement of both gas precursors to the flow axis, as well as of the newly nucleated nanoparticles, was attained by a coaxial argon (Ar) flow. The process was described in detail elsewhere [32]. The laser power and work pressure used were 55 W and 300 mbar, respectively, also presented in Table 1. The laser beam diameter was: φ = 2.0 mm.

In order to stabilize and functionalize the nanoparticles, the following protocol was used (ensuring the concentrations of 1 mg/mL NPs, 0.6 mg/mL CMCNa and 0.08 mg/mL chemotherapeutic drug (DOX)): The corresponding quantities for γ-Fe_2_O_3_ and CMCNa were mechanically mixed on an aluminum foil; at the same time, a glass vessel with the appropriate distilled water (pH = 5.5) load was placed in the ultrasound bath with the cooling system on. While in the ultrasound bath, a rotary mixer was introduced in the fluid and then the powders from the aluminium foil were slowly added. After all, the powder was introduced in the glass vessel, the mixer was removed, a cap was placed on the vessel, and it was left overnight in the ultrasound bath (59 kHz, 20 °C). The DOX powder was added at the end, and the final suspension was furthermore homogenised in the ultrasound bath for 1 h. The resulting suspensions γ-Fe_2_O_3__CMCNa_DOX and γ-Fe_2_O_3__DOX were centrifuged at 10 K rpm for 30 min, in order to eliminate the unanchored CMCNa and DOX. The solid deposit was then easily re-dispersed in distilled water maintaining the initial suspension volume using the same ultrasound bath for 30 min at 20 °C.

The resulted nanopowders were analyzed by the following methods: The phase composition and crystallinity were analized by X-ray diffraction (XRD) using a PANalytical X`Pert MPD theta-theta X-Ray diffraction apparatus with a Cu K α source (0.15418 nm); the morphology and structure were observed by transmission electron microscopy (TEM) and selected area electron diffraction (SAED) analysis, using a Philips CM 120ST (120 kV) Transmission Electron Microscope.

### 2.2. Spectroscopic Methods

The efficiency of DOX loading on γ-Fe_2_O_3_/γ-Fe_2_O_3__CMCNa was quantified by measuring the absorbance values with a Lambda 950 UV–vis-NIR spectrophotometer (PerkinElmer, Inc., Waltham, MA, US) and 5 mm for DOX and 0.1 mm for γ-Fe_2_O_3_/γ-Fe_2_O_3__CMCNa thickness optical quartz cuvettes (PerkinElmer, Inc., US). After solutions have been diluted from stock one, aiming to avoid saturation in the spectra, the absorption spectra were measured from 250 to 800 nm at room temperature (22–24 °C).

The efficiency of DOX loading was analysed also by laser induced fluorescence (LIF) home made set-up. The details of the experimental set-up were described elsewhere [24,25].

The laser excitation source consisted in the second harmonic generation (SHG) of a Nd:YAG laser (Continuum, US, Minilite II,) emitting at 532 nm, frequency 10 Hz. The laser pulse energies used in the experiments were smaller than 2.5 mJ to avoid a saturation effect of the processes and the DOX photodegradation. 

LIF signals were analyzed by a spectrograph (Acton Research, Acton, MA, US, model SpectraPro 2750) having a 150 tr/mm diffraction grating blazed at 500 nm. The detection was made by an iCCD (Princeton Instruments, Trenton, NJ, US, model PIMAX 1024 RB). The camera was triggered by a TTL laser-generated synchronizing pulse. The spectra were averaged on 1000 pulses.

The IR absorption study of the samples was performed by using a FTIR spectrometer (Thermo Fisher Scientific, Waltham, MA, US), model Nicolet iS50, resolution 4 cm^−1^. The Dox solution and NPs suspensions were dried on the surface of optical grade KRS-5 plates and the resulting film spectra were recorded in the mid IR range between 4000 and 400 cm^−1^. 

### 2.3. In Vitro Evaluation

#### 2.3.1. Cell Culture and Treatment

For in vitro tests two human breast cell lines were used: one cancerous MCF7 (ATCC HTB-22, LGC Standards, Manassas, VA, USA) and one normal MCF-12A (ATCC CRL-10782, Manassas, VA, USA). The MCF7 cells were cultured in DMEM medium (cat. no. 31600-083, Gibco, Dublin, Ireland) supplemented with 3.5 g/L glucose, 1.5 g/L NaHCO_3_, 1% antibiotics-antimycotics solution (A5955, Sigma-Aldrich, St. Louis, MO, USA) and 10% fetal bovine serum (FBS, cat. no. 10270-106, origin South America, Gibco, by Life Technologies, Carlsbad, CA, SUA), and the MCF-12A cells were cultured in DMEM: F12 medium (cat. no. 30-2006; Gibco, Dublin, Ireland) supplemented with 1.5 g/L NaHCO_3_, 20 µg/L human epidermal growth factor, 100 µg/L cholera toxin (C8050, Sigma, St. Louis, MO, USA), 10 mg/L bovine insulin, 500 µg/L hydrocortisone, 1% antibiotics-antimycotics solution (A5955, Sigma-Aldrich, St. Louis, MO, USA) and 10% FBS (cat. no. 10270-106, origin South America, Gibco, by Life Technologies, Carlsbad, CA, USA). 

For treatment, the samples in different concentrations, DOX (25, 50, 100, 250 ng/mL), free γ-Fe_2_O_3_ NPs/γ-Fe_2_O_3_ _CMCNa NPs (3.125, 6.25, 12.5, 31.25 µg/mL) and γ-Fe_2_O_3_ NPs/γ-Fe_2_O_3_ _CMCNa loaded with DOX (3.12–31.25 µg/mL NPs + 25–250 ng/mL DOX) were incubated with cells at 37 °C for 24 and 48 h. Untreated cells were used as control. Before incubation with cells, the NPs and doxorubicin suspensions were sterilized using UVC radiation for 1 h.

#### 2.3.2. Cell Morphology and Cytotoxicity Assessment

*a*. *Microscopic examination of cell morphology.* The morphology of tumor and normal breast cells was analyzed by optical microscopy after 24 and 48 h of incubation with the NPs and DOX suspensions. Different fields of cells were examined under an Olympus IX73 microscope (Olympus, Tokyo, Japan) equipped with a Hamamatsu ORCA-03G camera (A3472-06, Hamamatsu, Japan), and phase-contrast images were acquired using CellSens Dimension software (v1.11, Olympus).
*b*. *Live/Dead staining.* The cells were seeded in 24 well plates at a density of 3 × 10^4^ cells/mL and left to adhere overnight. After 24 and 48 h of treatment with NPs and DOX suspensions, the culture media was removed and replaced with a mix of calcein-AM and ethidium homodimer-1 solution following the manufacturer instructions of “LIVE/DEAD Viability/Cytotoxicity Kit for mammalian cells” (L3224, Invitrogen, Carlsbad, CA). The live (labeled in green) and dead (labeled in red) cells were analyzed under an Olympus IX73 fluorescence microscope (Olympus, Tokyo, Japan) equipped with a Hamamatsu ORCA-03G camera (A3472-06, Hamamatsu, Japan). The images were acquired using fluorescein isothiocyanate (FITC) and tri-rhodamine-isothiocyanate (TRITC) filters and CellSens Dimension software (v1.11, Olympus).
*c*. *MTT cell viability test.* The cells were seeded in 96 well plates at a density of 3 × 10^4^ cells/mL in 200 µL culture media. After exposure to DOX (25, 50, 100, 250 ng/mL) and NPs (3.125, 6.25, 12.5, 31.25 µg/mL) for 24 and 48 h, culture media was removed and replaced with 100 µL of 1 mg/mL MTT (3-(4,5-dimethylthiazol-2-yl)-2,5-diphenyltetrazolium bromide) for 2 h at 37 °C. The product formed (purple formazan) was solubilized with 150 µL isopropanol, and the absorbance of the samples was measured at 595 nm at a Flex Station 3 microplate reader (Molecular Devices, San Jose, CA, USA).
*d*. *Lactate dehydrogenase (LDH) assay.* The activity of LDH released in cell culture media as a result of cell membrane permeabilization was measured using the “Cytotoxicity Detection Kit (LDH)” (cat. no. 11644793001, Roche, Basel, Switzerland) in a 96 well plate. After exposure for 24 and 48 h to IONPs and DOX suspensions, 50 µL culture media of treated and control cells were incubated with 50 µL mix reaction (catalyst and dye solution) from the kit for 15 min at room temperature, in the dark. The absorbance of the samples was read at 490 nm using a microplate reader.

#### 2.3.3. Measurement of Reactive Oxygen Species (ROS) and Nitric Oxide (NO) Production

*a*. *DCF-DA intracellular ROS detection.* The MCF7 and MCF-12A cells were seeded in 96-well black sterile plates, clear bottom (165305, Thermo Scientific Nunc, Rochester, NY, USA), at a density of 3 × 10^4^ cells/mL. After cell adhesion, culture media was removed, and the cells were incubated with 100 µL of 50 µM H_2_DCFDA solution (2’,7’-dichlorodihydrofluorescein diacetate; D6883, Sigma-Aldrich, St. Louis, MO, USA) prepared in HBSS (Hank’s Balanced Salt Solution) for 1 h at 37 °C for entering cells. Further, the non-internalized H_2_DCFDA was removed, and the cells were treated with 6.25 and 12.5 µg/mL γ-Fe_2_O_3_ nanoparticles and, respectively, 50 and 100 ng/mL DOX. Upon generation of ROS, a fluorescent compound 2’,7’-dichlorofluorescein (DCF) was formed and read at 485 nm ex./520 nm em. after 4, 24 and 48 h of treatment. The level of ROS in treated samples was calculated in relation to the control sample and expressed in percentages.
*b*. *In vitro NO assay.* The level of NO released in culture media was measured to estimate the inflammatory potential of the suspensions on tumor and normal breast cells using Griess method. After the exposure of MCF7 and MCF-12A cells to 6.25 and 12.5 µg/mL NPs and, respectively, 50 and 100 ng/mL DOX for 24 and 48 h, 80 µL of culture media were mixed with 80 µL Griess reagent. The absorbance of the samples was measured at 540 nm. The NO concentration of the samples was determined using a NaNO_2_ standard curve (0–100 µM) and expressed as percentages relative to control.

#### 2.3.4. Statistical Analysis 

The results were calculated as an average of three replicates and represented relative to control (untreated cells). Statistical significance was calculated using Student’s test and a *p*-value < 0.05 was considered significant.

## 3. Results and Discussions

### 3.1. Characterization of Synthesized γ-Fe_2_O_3_ NPs

**Figure 2:**
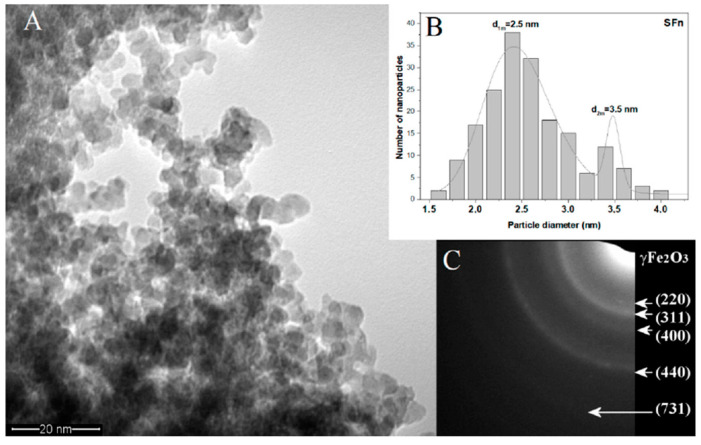
(**A**) TEM image of the γ-Fe_2_O_3_ powder after synthesis; (**B**) particle size distribution for γ-Fe_2_O_3_ with two maxima for particle diameter; (**C**) SAED pattern for γ-Fe_2_O_3_.

Figure 2A shows the TEM micrograph for sample γ-Fe_2_O_3_, and it can be observed that the nanoparticles present spherical shape and a tendency for agglomeration in small ramified chain-like aggregates. By a lognormal fit, two maxima for particle diameter distribution were found, *d_1m_ = 2.5* and *d_2m_ = 3.5 nm*. SAED analysis, presented as insert (2C), highlights the spinel structure of ɣ-Fe_2_O_3_/Fe_3_O_4_. These results are in agreement with the X-ray diffraction analysis pattern.

**Figure 3:**
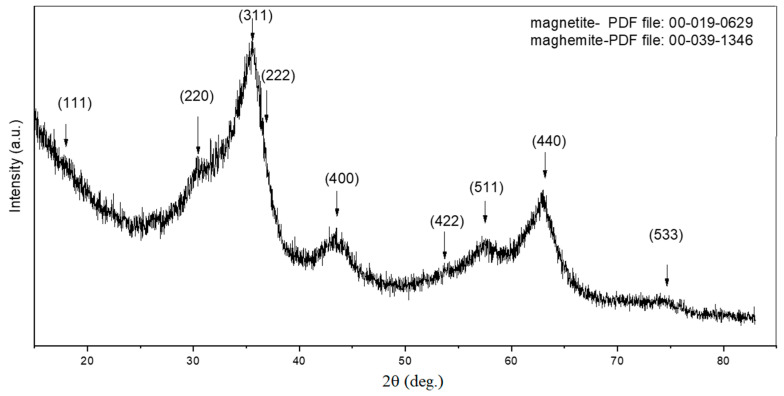
X-ray diffraction analysis of the synthesized γ-Fe_2_O_3_ NPs.

Figure 3 presents the XRD patterns for the sample γ-Fe_2_O_3_. The main crystalline phases detected were magnetite (Fe_3_O_4_) and maghemite (γ-Fe_2_O_3_). The rather broad peaks exhibited by the XRD patterns could be correlated with the decreased particle size, which was confirmed by further calculations. The mean crystallite dimension was 3.2 nm and was calculated with Scherrer equation based on FWHM (full width half maximum) for (440) peak, found at around 2θ = 63 degrees.

Previous papers were focused on structural and magnetic characterization of iron oxide NPs synthesized by laser pyrolysis in appropriate experimental conditions [32,33]. The magnetic hysteresis loops measured at different temperatures show a superparamagnetic feature for the as synthesized iron oxide NPs at room temperature with magnetic saturation of 32 emu/g and with almost zero remanent magnetization and coercivity above 150 K [33]. Only the iron oxide NPs with around 6 nm mean particle size show a small hysteresis loop at room temperature where the magnetic saturation grows up to 50 emu/g and the coercivity is less than 13 KA/m [32].

**Figure 4:**
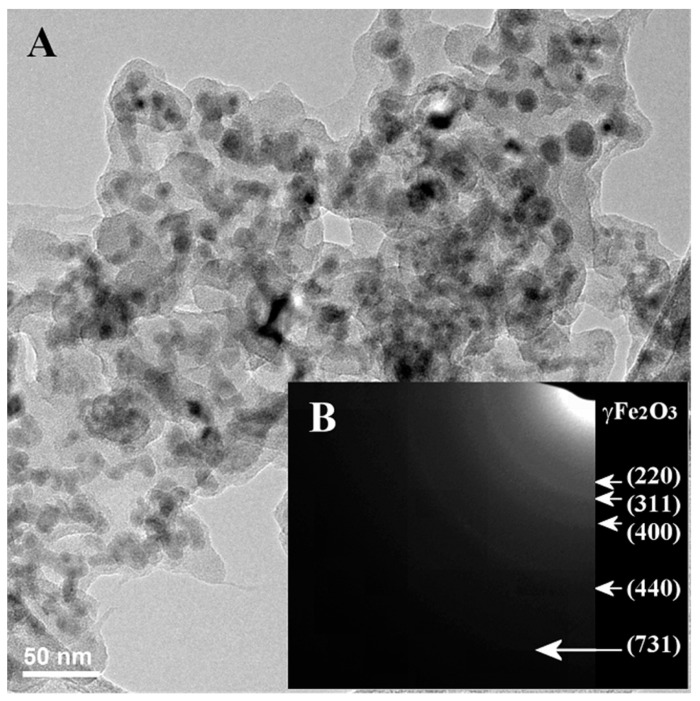
TEM (**A**) and SAED (**B**) images of γ-Fe_2_O_3__CMCNa NPs deposit extracted from an aqueous stabilized suspension.

A dry deposit from the γ-Fe_2_O_3__CMCNa NPs stabilized suspension was also investigated with TEM and SAED. As it can be seen in Figure 4A, the NPs-chains maintained the original feature, and they seem to be surrounded by a conformal polymeric-like shell with 3–7 nm thickness. Moreover, the SAED patterns (Figure 4B) resemble the original NPs (only γ-Fe_2_O_3_/Fe_3_O_4_ crystalline phase were revealed) which proves that after the suspension preparation, the iron oxide NPs keep their original crystallinity and suggests thus that the CMCNa stabilizing shell can preserve the crystalline magnetic phase of iron oxide (maghemite/magnetite). 

The attachment of CMCNa chains to iron oxide nanoparticles surface can be explained by the electrostatic attraction between the positive protonated Fe-OH^2+^ groups in slightly acidic aqueous medium and the negatively charged –CH_2_–COO– groups of the polyelectrolyte, reinforced also by multiple hydrogen bonds provided by hydroxyls from both oxidic surfaces and cellulose-derived chains.

### 3.2. Spectroscopic Analysis

**Figure 5:**
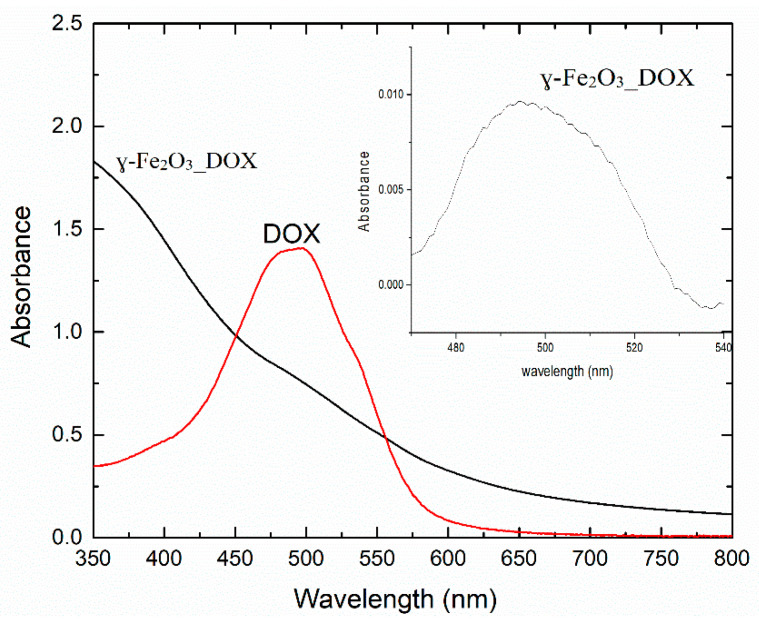
The absorption spectrum of DOX solution (0.02 mg/mL) and the γ-Fe_2_O_3__DOX suspension in distilled water (1:10 dilution).

The absorption spectrum for the γ-Fe_2_O_3__DOX sample was compared to the absorption spectrum for a solution of DOX in distilled water at 0.02 mg/mL (Figure 5). The γ-Fe_2_O_3__DOX suspension was diluted 1:10 to avoid saturation of the absorption signal. In the inset of Figure 5, it is shown the spectrum of the DOX conjugated NPs suspension after background subtraction.

From the absorbance values corresponding to the absorption peak of Dox located at 500 nm for the suspension of the NPs functionalised with DOX and the corresponding absorbance of DOX at a known concentration of 0.02 mg/mL, we determined the amount of DOX loaded on the NPs. A concentration of 0.8 µg/mL in the 1:10 dilution of γ-Fe_2_O_3__DOX suspension has been found, this corresponding to a loading efficiency of 10%. The samples containing CMCNa in the suspension were difficult to analyze by absorption spectroscopy, due to the absorption band of CMCNa which hinders the subtraction of the DOX absorption band intensity in order to estimate the loading efficiency. 

**Figure 6:**
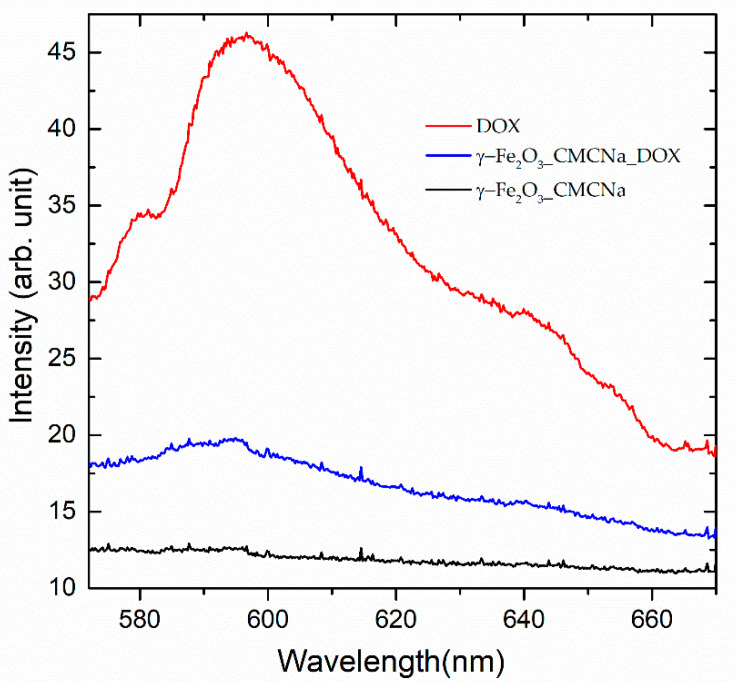
Fluorescence spectra excited with 532 nm at 2.5 mJ for a solution of Dox in distilled water (0.02 mg/mL), γ-Fe_2_O_3__CMCNa and γ-Fe_2_O_3__CMCNa_DOX suspensions in distilled water.

This suspension containing CMCNa was analyzed by LIF. The emission spectra excited with 532 nm at 2.5 mJ for Dox solution (0.02 mg/mL), γ-Fe_2_O_3__CMCNa and γ-Fe_2_O_3__CMCNa_DOX suspensions are shown in Figure 6. The NPs samples used were 1:10 dilutions of the stock suspensions. 

From the fluorescence signals intensities at the maximum of the emission band of DOX placed at 600 nm, we can extract the amount of DOX loaded on the NPs. In this case, we estimated a value of approximately 8% from the initial DOX quantity used at mixture preparation. 

In both, absorption and fluorescence spectra, it can be noticed besides the main monomer bands (495 and 596 nm), the ones for dimers (537 and 641 nm) which can be formed by aggregation [34].

Maybe, due to the fact that Dox forms aggregates, the amount of DOX loaded on the NPs is rather small. The adsorption of DOX on NPs is proved by the shift of the fluorescence emission peak to shorter wavelength (593 nm) for the conjugates γ-Fe_2_O_3__CMCNa_DOX compared to simple Dox solutions (596 nm).

**Figure 7:**
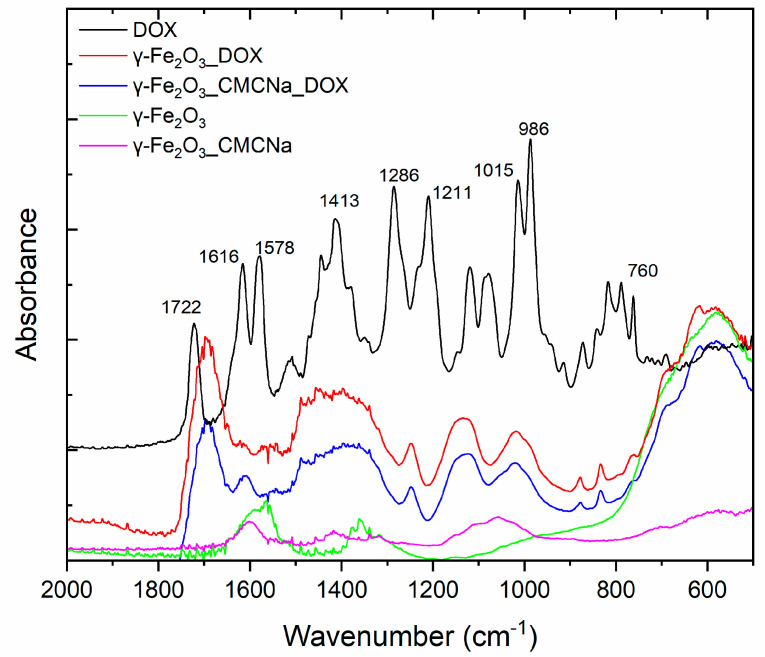
FTIR spectra for DOX, γ-Fe_2_O_3__DOX, γ-Fe_2_O_3__CMCNa_DOX, γ-Fe_2_O_3__CMCNa and γ-Fe_2_O_3_ samples.

The samples were analysed also by FTIR spectroscopy to emphasize the conjugation of NPs with the drug, via IR bands of the specific functional groups. In Figure 7, the IR absorption spectra for DOX, γ-Fe_2_O_3__DOX, γ-Fe_2_O_3__CMCNa_DOX, γ-Fe_2_O_3__CMCNa and γ-Fe_2_O_3_ samples are shown. It can be noticed the Dox specific bands present in the spectra of conjugated NPs: C-C bending at 1413 cm^−1^, C–O, C–H deformation vibrations at 1015 cm^−1^ and respectively 986 cm^−1^. The adsorption of Dox on NPs is evidenced by the C=O stretching vibration shift from 1722 cm^−1^ to 1688 cm^−1^ and C–O–C stretching vibration slightly shift from 1286 cm^−1^ to 1251 cm^−1^. The attachment of DOX to Np can occur via the interaction of –NH_2_ and –OH groups of DOX with –OH groups of absorbed water on Np through hydrogen bonding [35]. In support of this, N-H vibrations present in Dox spectrum at 1616 cm^−1^ and 760 cm^−1^ are clearly diminished in conjugated samples spectra. On the other side, the 1601 cm^−1^ H–O–H bending vibration of adsorbed water present in the spectra of iron nanoparticles disappears in the spectra of γ-Fe2O3_ DOX.

The presence of polyanionic CMCNa chains wrapped on the Nps can enhance the adsorption of DOX due to their amino group electrostatic attraction towards carboxy negative groups of CMCNa, doubled by the possible formation of some hydrogen bonds between the hydroxyls groups of polymer and those from DOX [36].

### 3.3. In Vitro Biological Evaluation

#### 3.3.1. Anticancer Cell Efficiency

The cytotoxicity of the DOX, γ-Fe_2_O_3_, γ-Fe_2_O_3__CMCNa, γ-Fe_2_O_3__DOX and γ-Fe_2_O_3__CMCNa_DOX suspensions to MCF7 and MCF-12A cells was estimated and confirmed after 24 and 48 h of exposure by three tests: MTT, Live/Dead, and LDH assays. Morphological alterations were also observed and presented by optical microscopy images.

**Figure 8:**
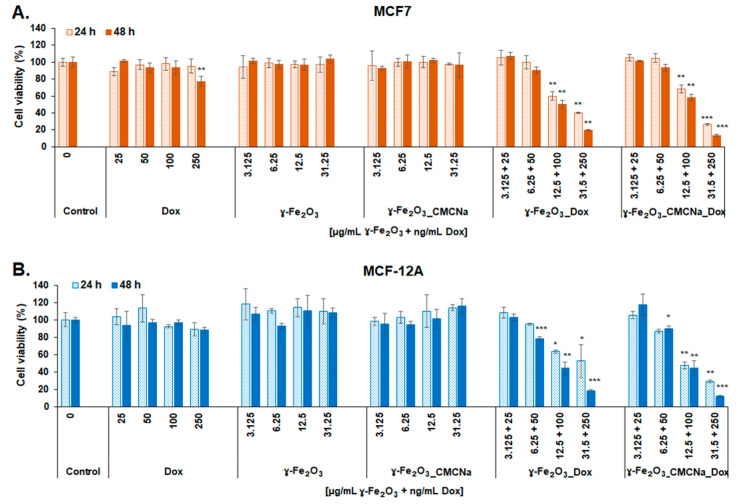
MTT test. Viability of MCF7 (**A**) and MCF-12A (**B**) cells after 24 and 48 h incubation with DOX, γ-Fe_2_O_3_, γ-Fe_2_O_3__CMCNa, γ-Fe_2_O_3__DOX and γ-Fe_2_O_3__CMCNa_DOX suspensions (mean ± SD, *n* = 3). The statistical significance of results was noted with asterisk (*) as follow: * *p* < 0.05 (weakly significant); ** *p* < 0.01 (moderately significant); *** *p* < 0.001 (highly significant).

Figure 8 representing MTT evaluation shows that both γ-Fe_2_O_3__DOX and γ-Fe_2_O_3__CMCNa_DOX induced a significant decrease of cell viability at doses higher than 12.5 µg/mL γ-Fe_2_O_3_ and respectively 100 ng/mL DOX most likely caused by a change in the cell metabolic activity or enzyme activity in the mitochondrial respiratory chain. The cytotoxicity was time- and dose-dependent for both human cell lines. A decrease by 50% of MCF7 cell viability was obtained after 48 h incubation with γ-Fe_2_O_3__DOX (in a dose of 12.5 µg/mL γ-Fe_2_O_3_ + 100 ng/mL DOX) and only by 42% with γ-Fe_2_O_3__CMCNa_DOX compared to control (unexposed cells). For MCF-12A cells, the decline of cell viability was closely similar to the one for MCF7 cells registering a decrease of 56% for the same conditions. For free DOX, γ-Fe_2_O_3_ or γ-Fe_2_O_3__CMCNa, the results indicated no decrease of MCF7 and MCF-12A cell viability during the experiment period in a concentration range between 3.125 and 31.25 µg/mL γ-Fe_2_O_3_ NPs and 25 and 250 ng/mL DOX, suggesting high biocompatibility for NPs but no cytotoxicity of DOX in this range of concentrations. Only a dose of 250 ng/mL free DOX caused a slight decrease of MCF7 cells viability by 22.6% compared to control cells.

According to literature, the half-maximal inhibitory concentration (IC_50_) for DOX was obtained at a concentration between 0.3 and 4.6 µg/mL after 48 h exposure of different human breast cells [37]. By comparison, our data showed that cytotoxicity of DOX increases significantly when conjugated with γ-Fe_2_O_3_ and γ-Fe_2_O_3__CMCNa; a 50% reduction of cell viability was obtained with a concentration around 0.1 µg/mL. 

**Figure 9:**
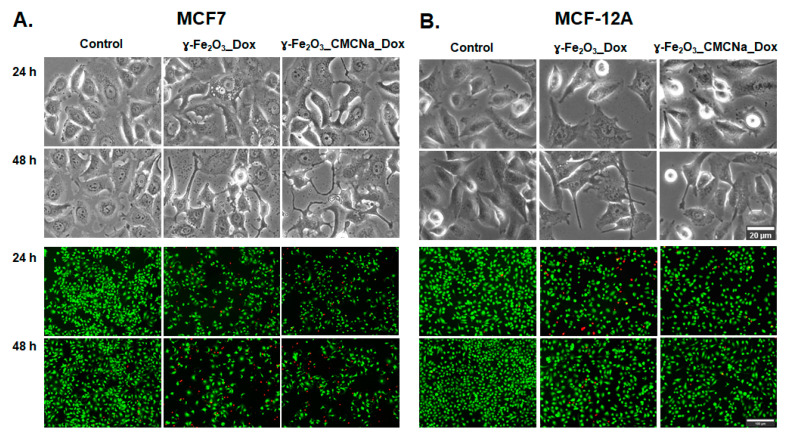
Cell morphology (bright-field images) and Live/Dead staining (fluorescence images) for MCF7 (**A**) and MCF-12A (**B**) cells after 24 and 48 h incubation with a dose of 12.5 µg/mL γ-Fe_2_O_3_ + 100 ng/mL DOX γ-Fe_2_O_3__DOX and γ-Fe_2_O_3__CMCNa_DOX. Scale bar for bright-field images = 20 µm; scale bar for fluorescence images = 100 µm.

The anticancer efficiency of nanosuspensions conjugated with DOX was also confirmed by microscopy images of cells (Figure 9, bright-field images). The morphological observations revealed significant alterations of MCF7 cells compared to control cells. While control cells showed intact cell membrane, the treated MCF7 cells were characterized by cell shrinkage, extensions of the cell membrane, presence of large vacuoles in the cytoplasm and apoptotic bodies (cell fragments) when exposed to 12.5 µg/mL γ-Fe_2_O_3_ + 100 ng/mL DOX γ-Fe_2_O_3__DOX and γ-Fe_2_O_3__CMCNa_DOX, which might indicate the occurrence of apoptosis. However, we found that the morphological changes in MCF-12A normal breast cells were less severe. 

Consistent with the findings from the morphological examination, the Live/Dead assay results showed a clear decrease in the number of MCF7 live cells (green fluorescence) concomitant with an increase in the number of dead cells (red fluorescence) for both γ-Fe_2_O_3__DOX and ɣ-Fe_2_O_3__CMCNa_DOX compared to control cells. In contrast, the number of live non-tumoral breast cells was slightly higher than of tumoral cells after 48 h of exposure. In addition, the Live/Dead images might suggest besides apoptotic death, a decrease in MCF7 cell proliferation because fewer cells were noticed in the acquired images. The antiproliferative effect of DOX on cancer cells was already demonstrated but the exact mechanism that inhibits cell proliferation is not yet fully understood [38].

**Figure 10:**
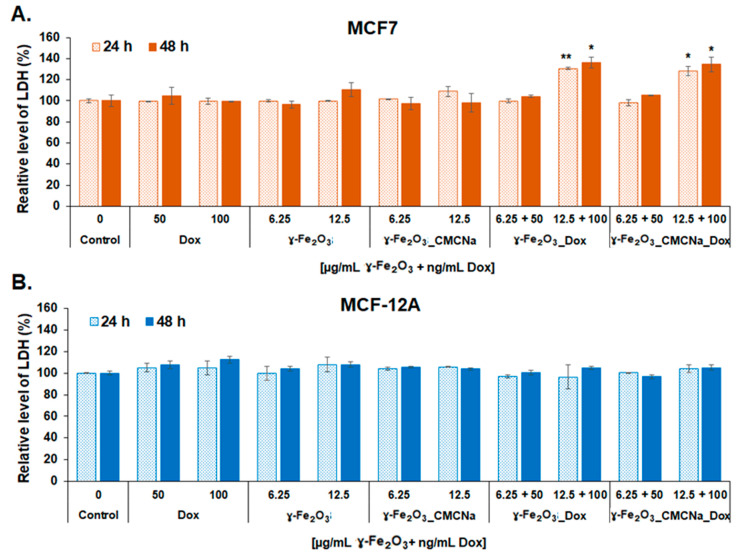
LDH leakage after membrane damage of MCF7 (**A**) and MCF-12A (**B**) cells incubated with DOX, γ-Fe_2_O_3_, γ-Fe_2_O_3__CMCNa, γ-Fe_2_O_3__DOX and γ-Fe_2_O_3__CMCNa_DOX suspensions for 24 and 48 h (mean ± SD, *n* = 3) The statistical significance of results was noted with asterisk (*) as follow: * *p* < 0.05 (weakly significant); ** *p* < 0.01 (moderately significant).

After treatment, the LDH released in culture media was also measured to estimate the cell membrane damage after 24 and 48 h. As shown in Figure 10, loss of cell membrane integrity was present only for MCF7 cells treated with 12.5 µg/mL γ-Fe_2_O_3_ + 100 ng/mL DOX γ-Fe_2_O_3__DOX and γ-Fe_2_O_3__CMCNa_DOX, where the level of LDH increased significantly with a maximum of 36% and 34%, respectively, after 48 h of exposure compared to control. However, no modifications of LDH level were observed for MCF-12A cells, which suggests that normal breast cells succeeded in keeping their membrane intact. This result was not previously reported and is an important finding, which reveals opportunities for new therapeutic approaches to tackle this disease.

Membrane extension, cell shrinkage and apoptotic bodies are features of apoptosis while the loss of cell membrane integrity is a feature of necrosis. Thus, our results might suggest a possible combination of more types of cell death induced in MCF7 cells by DOX-nanoconjugates, which could increase the anticancer efficiency by targeting more cell pathways.

Previous studies showed that different doses of DOX activate different regulatory mechanisms to induce either apoptosis, necrosis or cell death through mitotic catastrophe [39,40]. In vitro, free DOX has been shown to induce apoptosis via the activation of caspases and disruption of mitochondrial membrane potential. For example, Nestal de Moraes et al. demonstrated that DOX inhibited cell viability and induced DNA fragmentation and activation of caspases−3, −7 and −9 in the breast cancer-derived cell lines MCF7 and MDA-MB-231 [41]. However, more recently, it was reported the absence of active caspase 3 and cleavage of PARP 1 in MCF7 cells treated with DOX for 48 h [42].

By combining DOX with IONPs, it was possible to increase the drug intracellular concentration by escaping the endosomal retention and thus overcome resistance [43] and even cross the blood–brain barrier [44].

#### 3.3.2. Oxidative and Inflammatory Potential

**Figure 11:**
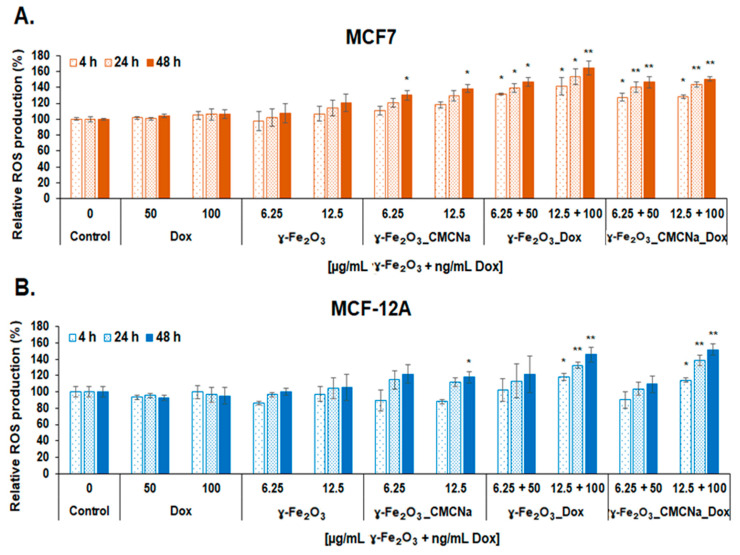
ROS generation in MCF7 (**A**) and MCF-12A (**B**) cells after 4, 24 and 48 h incubation with Dox, γ-Fe_2_O_3_, γ-Fe_2_O_3__CMCNa, γ-Fe_2_O_3__DOX and γ-Fe_2_O_3__CMCNa_DOX suspensions (mean ± SD, *n* = 3). The statistical significance of results was noted with asterisk (*) as follow: * *p* < 0.05 (weakly significant); ** *p* < 0.01 (moderately significant).

The level of ROS was analyzed to evaluate the capacity of DOX-nanoconjugates to induce oxidative stress in tumor and normal cells. As seen in Figure 11, the treatment with nanoconjugates caused a time-dependent increase of intracellular ROS in both MCF7 and MCF-12A cells. The level of ROS was higher in tumoral cells compared with normal cells. Overproduction of ROS was recorded for both doses of DOX-nanoconjugates in MCF7 cells and only for the higher dose in MCF-12A cells. More importantly, the results showed a higher production of ROS in MCF7 exposed to γ-Fe_2_O_3__DOX compared to γ-Fe_2_O_3__CMCNa_DOX for the dose of 12.5 µg/mL γ-Fe_2_O_3_ + 100 ng/mL DOX starting with 4 h. No significant increase of ROS level was observed for free γ-Fe_2_O_3__DOX but only for γ-Fe_2_O_3__CMCNa after 48 h. We think that the negatively charged CMCNa-coating of γ-Fe2O3 NPs might attract a large number of protein molecules on the surface of NPs that possibly hinder the DOX cytotoxicity. Cancer cells are known to acidify their environment and, consequently, serum proteins tend to have positive charges which interact with the functional carboxymethyl groups of CMCNa and surround the NPs forming a biocorona of proteins.

It is well known that the generation of free radicals is one of the mechanisms by which DOX induces its anticancer effect. DOX accumulates in mitochondria by specifically binding to the phospholipid cardiolipin which is found abundantly in the inner mitochondrial membrane. At this level, the membrane perturbation inhibits complex I and complex II, disrupting the electron transport chain leading to ROS production. Besides this, it was demonstrated that DOX can also generate ROS through other mechanisms. For example, DOX can directly interact with iron to form reactive anthracycline-iron complexes resulting in an iron cycling between Fe^3+^ and Fe^2+^ associated with ROS production including the high-toxic hydroxyl radical (OH•) by the Fenton and Haber–Weiss reactions, thus altering iron homeostasis [45].

Previous studies reported that DOX also increases nitric oxide synthase (NOS) activity and expression and, by direct binding to this enzyme, leading to nitric oxide (NO) production [45,46].

**Figure 12:**
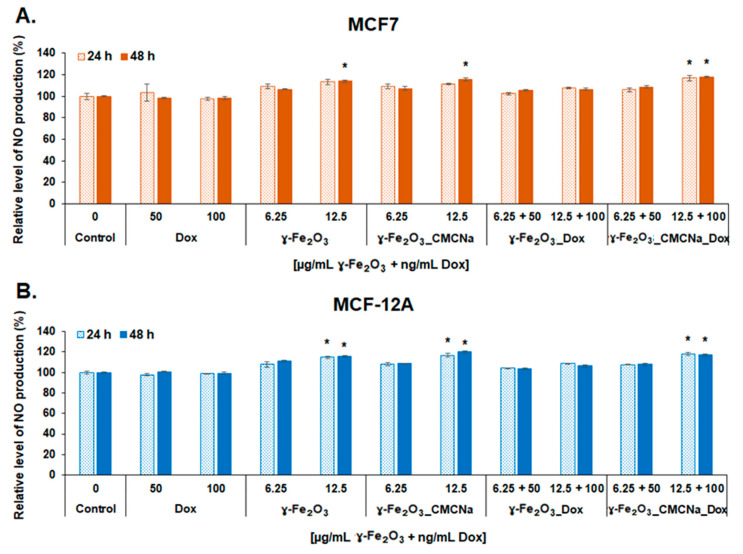
Level of NO release in culture media of MCF7 (**A**) and MCF-12A (**B**) cells after 24 and 48 h incubation with DOX, γ-Fe_2_O_3_, γ-Fe_2_O_3__CMCNa, γ-Fe_2_O_3__DOX and γ-Fe_2_O_3__CMCNa_DOX suspensions (mean ± SD, *n* = 3). The statistical significance of results was noted with asterisk (*) as follow: * *p* < 0.05 (weakly significant).

In this study, the NO level produced in culture media of tumoral and normal breast cells seems to be more a result of γ-Fe_2_O_3_ NPs exposure and less of DOX one. As seen in Figure 12, exposure to simple γ-Fe_2_O_3_ and γ-Fe_2_O_3__CMCNa in a dose of 12.5 µg/mL resulted in a statistically significant elevation of NO production starting with 24 h for normal cells and 48 h for tumoral cells suggesting a slight inflammatory potential of bare NPs. However, an increase of NO production (no more than 17% over control) was also detected for both cell lines treated with γ-Fe_2_O_3__CMCNa_DOX at a dose of 12.5 µg/mL γ-Fe_2_O_3_ + 100 ng/mL DOX but the level was similar to the one found in cells exposed only to free NPs. These results suggested that DOX had no contribution to NO production. Interestingly, exposure of cells to γ-Fe_2_O_3__DOX did not cause any change in NO level at none of the concentrations, which made us to conclude that the combination with DOX might decrease the cytotoxicity of bare γ-Fe_2_O_3_.

## 4. Conclusions

The present study demonstrated the efficiency of γ-Fe_2_O_3_ NPs loaded with DOX on the decrease of viability of breast cancer cells. The NPs were synthesized by laser pyrolysis, and their nanometric size and crystallinity were confirmed by XRD and TEM analyses.

The NPs were stabilized with CMCNa and loaded with DOX. The loading efficiency was estimated by using absorption and fluorescence spectroscopy, and it was about 10%. The conjugation of NPs with the drug was evidenced by FTIR spectroscopy.

According to the biological results obtained in the current investigation, both utilized DOX-nanoconjugates present a good anticancer potential demonstrated through the inhibition of tumoral cell proliferation, disruption of the cellular membrane, induction of cell death, and reduced effects on normal breast cells. However, we also found that the coating of γ-Fe_2_O_3_ with CMCNa might hinder the DOX-ROS production and thus its cytotoxicity in comparison to uncoated γ-Fe_2_O_3_. Comparing with previous works, this study presents not only the cytotoxic effect of NPs but also their oxidative and inflammatory potential. Further studies are needed to explore the cytotoxic potential of these nanoconjugates in vivo.

## Figures and Tables

**Table 1:** Experimental data.

Sample	D-C_2_H_4_/Fe(CO)_5_	D-Synth. Air	D-Ar conf	D-Ar windows	*p*	*p*_laser_
m.u.	sccm	sccm	sccm	sccm	mbar	W
γ-Fe_2_O_3_	100/20	100	2000	300	300	55/53

## References

[1] Anastasiadi Z., Lianos G.D., Ignatiadou E., Harissis H.V., Mitsis M. (2017). Breast cancer in young women: An overview. Updates Surg..

[2] Libson S., Lippman M. (2014). A review of clinical aspects of breast cancer. Int. Rev. Psychiatry.

[3] Preventing Cancer. http://www.who.int/cancer/prevention/diagnosis-screening/breast-cancer/en/.

[4] Su Y.L., Hu S.H. (2018). Functional Nanoparticles for Tumor Penetration of Therapeutics. Pharmaceutics.

[5] Caron J., Nohria A. (2018). Cardiac Toxicity from Breast Cancer Treatment: Can We Avoid This?. Curr. Oncol. Rep..

[6] Conte P.F., Gennari A., Landucci E., Orlandini C. (2000). Role of Epirubicin in Advanced Breast Cancer. Clin. Breast Cancer.

[7] Marinello J., Delcuratolo M., Capranico G. (2018). Anthracyclines as Topoisomerase II Poisons: From Early Studies to New Perspectives. IJMS.

[8] Rivankar S. (2014). An overview of doxorubicin formulations in cancer therapy. J. Can. Res. Ther..

[9] Shafei A., El-Bakly W., Sobhy A., Wagdy O., Reda A., Aboelenin O., Marzouk A., El Habak K., Mostafa R., Ali M.A. (2017). A review on the efficacy and toxicity of different doxorubicin nanoparticles for targeted therapy in metastatic breast cancer. Biomed. Pharmacother..

[10] Anghel N., Herman H., Balta C., Rosu M., Stan M., Nita D., Ivan A., Galajda Z., Ardelean A., Dinischiotu A. (2017). Acute cardiotoxicity induced by doxorubicin in right ventricle is associated with increase of oxidative stress and apoptosis in rats. Histol. Histopathol..

[11] Hanusova V., Skalova L., Kralova V., Matouskova P. (2015). Potential Anti-cancer Drugs Commonly Used for Other Indications. CCDT.

[12] Lloyd-Parry O., Downing C., Aleisaei E., Jones C., Coward K. (2018). Nanomedicine applications in women’s health: State of the art. IJN.

[13] Albanese A., Tang P.S., Chan W.C.W. (2012). The Effect of Nanoparticle Size, Shape, and Surface Chemistry on Biological Systems. Annu. Rev. Biomed. Eng..

[14] Jain R.K., Stylianopoulos T. (2010). Delivering nanomedicine to solid tumors. Nat. Rev. Clin. Oncol..

[15] Perrault S.D., Walkey C., Jennings T., Fischer H.C., Chan W.C.W. (2009). Mediating Tumor Targeting Efficiency of Nanoparticles Through Design. Nano Lett..

[16] Mu Q., Wang H., Zhang M. (2017). Nanoparticles for imaging and treatment of metastatic breast cancer. Expert Opin. Drug Deliv..

[17] Shabestari Khiabani S., Farshbaf M., Akbarzadeh A., Davaran S. (2017). Magnetic nanoparticles: Preparation methods, applications in cancer diagnosis and cancer therapy. Artif. Cells Nanomed. Biotechnol..

[18] Jordan A., Scholz R., Wust P., Fähling H., Felix R. (1999). Magnetic fluid hyperthermia (MFH): Cancer treatment with AC magnetic field induced excitation of biocompatible superparamagnetic nanoparticles. J. Magn. Magn. Mater..

[19] Martinkova P., Brtnicky M., Kynicky J., Pohanka M. (2018). Iron Oxide Nanoparticles: Innovative Tool in Cancer Diagnosis and Therapy. Adv. Healthc. Mater..

[20] Huber D. (2005). Synthesis, Properties, and Applications of Iron Nanoparticles. Small.

[21] Ali A., Zafar H., Zia M., ul Haq I., Phull A.R., Ali J.S., Hussain A. (2016). Synthesis, characterization, applications, and challenges of iron oxide nanoparticles. NSA.

[22] Dadfar S.M., Roemhild K., Drude N.I., von Stillfried S., Knüchel R., Kiessling F., Lammers T. (2019). Iron oxide nanoparticles: Diagnostic, therapeutic and theranostic applications. Adv. Drug Deliv. Rev..

[23] Quan Q., Xie J., Gao H., Yang M., Zhang F., Liu G., Lin X., Wang A., Eden H.S., Lee S. (2011). HSA Coated Iron Oxide Nanoparticles as Drug Delivery Vehicles for Cancer Therapy. Mol. Pharm..

[24] Xie J., Lee S., Chen X. (2010). Nanoparticle-based theranostic agents. Adv. Drug Deliv. Rev..

[25] Zhu L., Zhou Z., Mao H., Yang L. (2017). Magnetic nanoparticles for precision oncology: Theranostic magnetic iron oxide nanoparticles for image-guided and targeted cancer therapy. Nanomedicine.

[26] Yu M.K., Jeong Y.Y., Park J., Park S., Kim J.W., Min J.J., Kim K., Jon S. (2008). Drug-Loaded Superparamagnetic Iron Oxide Nanoparticles for Combined Cancer Imaging and Therapy In Vivo. Angew. Chem. Int. Ed..

[27] Jain T.K., Richey J., Strand M., Leslie-Pelecky D.L., Flask C.A., Labhasetwar V. (2008). Magnetic nanoparticles with dual functional properties: Drug delivery and magnetic resonance imaging. Biomaterials.

[28] Plichta Z., Kozak Y., Panchuk R., Sokolova V., Epple M., Kobylinska L., Jendelová P., Horák D. (2018). Cytotoxicity of doxorubicin-conjugated poly[*N*-(2-hydroxypropyl)methacrylamide]-modified γ-Fe_2_O_3_ nanoparticles towards human tumor cells. Beilstein J. Nanotechnol..

[29] Plichta Z., Horák D., Mareková D., Turnovcová K., Kaiser R., Jendelová P. (2020). Poly[*N*-(2-hydroxypropyl)methacrylamide]-Modified Magnetic γ-F_2_O_3_ Nanoparticles Conjugated with Doxorubicin for Glioblastoma Treatment. ChemMedChem.

[30] Li S., Zhang R., Wang D., Feng L., Cui K. (2020). Synthesis of hollow maghemite (-Fe_2_O_3_) particles for magnetic field and pH-responsive drug delivery and lung cancer treatment. Ceram. Int..

[31] Bomatí-Miguel O., Zhao X.Q., Martelli S., Di Nunzio P.E., Veintemillas-Verdaguer S. (2010). Modeling of the laser pyrolysis process by means of the aerosol theory: Case of iron nanoparticles. J. Appl. Phys..

[32] Morjan I., Alexandrescu R., Dumitrache F., Birjega R., Fleaca C., Soare I., Luculescu C.R., Filoti G., Kuncer V., Vekas L. (2010). Iron oxide-based nanoparticles with different mean sizes obtained by the laser pyrolysis: Structural and magnetic properties. J. Nanosci. Nanotechnol..

[33] Greculeasa S.G., Palade P., Schinteie G., Leca A., Dumitrache F., Lungu I., Prodan G., Kuncser A., Kuncser V. (2020). Tuning structural and magnetic properties of Fe oxide nanoparticles by specific hydrogenation treatments. Sci. Rep..

[34] Motlagh N.S.H., Parvin P., Ghasemi F., Atyabi F. (2016). Fluorescence properties of several chemotherapy drugs: Doxorubicin, paclitaxel and bleomycin. Biomed. Opt. Express.

[35] Kayal S., Ramanujan R.V. (2010). Doxorubicin loaded PVA coated iron oxide nanoparticles for targeted drug delivery. Mater. Sci. Eng. C.

[36] Rana S., Gallo A., Srivastava R.S., Misra R.D.K. (2007). On the suitability of nanocrystalline ferrites as a magnetic carrier for drug delivery: Functionalization, conjugation and drug release kinetics. Acta Biomater..

[37] Wen S., Su S., Liou B., Lin C., Lee K. (2018). Sulbactam-enhanced cytotoxicity of doxorubicin in breast cancer cells. Cancer Cell Int..

[38] Denard B., Lee C., Ye J. (2012). Doxorubicin blocks proliferation of cancer cells through proteolytic activation of CREB3L1. eLife.

[39] Eom Y.W., Kim M.A., Park S.S., Goo M.J., Kwon H.J., Sohn S., Kim W.H., Yoon G., Choi K.S. (2005). Two distinct modes of cell death induced by doxorubicin: Apoptosis and cell death through mitotic catastrophe accompanied by senescence-like phenotype. Oncogene.

[40] Shin H.J., Kwon H.K., Lee J.H., Gui X., Achek A., Kim J.H., Choi S. (2015). Doxorubicin-induced necrosis is mediated by poly-(ADP-ribose) polymerase 1 (PARP1) but is independent of p53. Sci. Rep..

[41] Nestal de Moraes G., Vasconcelos F.C., Delbue D., Mognol G.P., Sternberg C., Viola J.P.B., Maia R.C. (2013). Doxorubicin induces cell death in breast cancer cells regardless of Survivin and XIAP expression levels. Eur. J. Cell Biol..

[42] Kumar A., Patel S., Bhatkar D., Sharma N.K. (2019). A novel method to detect intracellular metabolite alterations in MCF-7 cells by doxorubicin induced cell death. bioRxiv.

[43] Kievit F.M., Wang F.Y., Fang C., Mok H., Wang K., Silber J.R., Ellenbogen R.G., Zhang M. (2011). Doxorubicin loaded iron oxide nanoparticles overcome multidrug resistance in cancer in vitro. J. Control. Release.

[44] Norouzi M., Yathindranath V., Thliveris J.A., Kopec B.M., Siahaan T.J., Miller D.W. (2020). Doxorubicin-loaded iron oxide nanoparticles for glioblastoma therapy: A combinational approach for enhanced delivery of nanoparticles. Sci. Rep..

[45] Gorini S., De Angelis A., Berrino L., Malara N., Rosano G., Ferraro E. (2018). Chemotherapeutic Drugs and Mitochondrial Dysfunction: Focus on Doxorubicin, Trastuzumab, and Sunitinib. Oxidative Med. Cell. Longev..

[46] Asensio-López M.C., Soler F., Pascual-Figal D., Fernández-Belda F., Lax A. (2017). Doxorubicin-induced oxidative stress: The protective effect of nicorandil on HL-1 cardiomyocytes. PLoS ONE.

